# Rural age‐friendly ecosystems for older adults: An international scoping review with recommendations to support age‐friendly communities

**DOI:** 10.1002/hsr2.1241

**Published:** 2023-05-05

**Authors:** Daniel Liebzeit, Anna Krupp, Jacinda Bunch, Shalome Tonelli, Emily Griffin, Sarah McVeigh, Nai‐Ching Chi, Saida Jaboob, Lynn Nakad, Alicia I. Arbaje, Harleah Buck

**Affiliations:** ^1^ The University of Iowa College of Nursing Iowa City Iowa USA; ^2^ Department of Medicine, Division of Geriatric Medicine and Gerontology, Center for Transformative Geriatric Research Johns Hopkins University School of Medicine Baltimore Maryland USA; ^3^ Department of Health Policy and Management Johns Hopkins University Bloomberg School of Public Health Baltimore Maryland USA; ^4^ Armstrong Institute Center for Health Care Human Factors Johns Hopkins University School of Medicine Baltimore Maryland USA

**Keywords:** community health services, delivery of healthcare, healthy aging, rural health services, rural population

## Abstract

**Background and Aims:**

The population of older adults in rural areas is rising, and they experience higher rates of poverty and chronic illness, have poorer health behaviors, and experience different challenges than those in urban areas. This scoping review seeks to (1) map the state of the science of age‐friendly systems in rural areas regarding structural characteristics, processes for delivering age‐friendly practices, and outcomes of age‐friendly systems, (2) analyze strengths, weakness, opportunities, and threats of age‐friendly system implementation, and (3) make person, practice, and policy‐level recommendations to support active aging and development of age‐friendly communities.

**Methods:**

An international scoping review was conducted of articles that used age‐friendly framing, had a sample age of 45 years of age or older, self‐identified as rural, and reported empiric data. Searches were conducted in PubMed, CINAHL, AgeLine, PsychINFO, EMBASE, Scopus, and Academic Search Elite on October 26, 2021, and rerun March 10, 2023. Data were charted across three analytic layers: socioecological model, Donabedian's framework, and SWOT analysis.

**Results:**

Results reveal limited data on outcomes relevant to organizations, such as return on investment or healthcare utilization. While the SWOT analysis revealed many strengths of age‐friendly systems, including their impact on persons' outcomes, it also revealed several weaknesses, threats, and gaps. Namely, age‐friendly systems have weaknesses due to reliance on trained volunteers and staff, communication, and teamwork. System‐level threats include community and health system barriers, and challenges in poor/developing areas.

**Conclusions:**

While age‐friendly systems in this review were heterogeneous, there is an opportunity to focus on unifying elements including the World Health Organization age‐friendly cities framework or 4Ms framework for age‐friendly care. Despite the many benefits of age‐friendly systems, we must acknowledge limitations of the evidence base, pursue opportunities to examine organizational metrics to support implementation and sustainability of age‐friendly systems, and leverage improvements in age‐friendliness at a community level.

## 1 INTRODUCTION

1.1

Age‐friendly as a concept has evolved from its beginnings in the early years of the 21st century. In 2006, the World Health Organization developed an action plan focused on improving the lives of older adults (age 65 and older) across the globe by identifying how communities could become “age‐friendly” and encourage active aging, the process of optimizing opportunities for health, participation, and security to enhance quality of life as people age.^1^, ^2^ This led to a worldwide movement and ecosystem encompassing age‐friendly policies, public health systems, universities, cities, states, and health systems.^3^ The importance of age‐friendliness and the growing body of literature surrounding age‐friendly communities is evidenced by a recent special issue in a major gerontology journal specifically focused on examining “age‐friendliness” at the intersection of person and environment, aging in place, and measurement.^4^ The issue, international in scope, is an important representation of the state of the science related to real‐world age‐friendly initiatives. One omission in the special issue and the extant literature is a thoughtful global synthesis of the rural age‐friendly empiric literature. Current reviews of the rural age‐friendly literature are either outdated and contain a smaller number of studies,^5^, ^6^ or are more recent but focused on a single country.^7^ A more recent review does focus on application of individual concepts from the WHO age‐friendly cities framework (e.g., healthcare or housing for older adults), but omits projects that truly apply an age‐friendly framework.^8^

Examining age‐friendly systems, systems (cities, communities, or organizations) which support active aging, engagement/participation, and well‐being of older persons and their caregivers, in rural areas is critically important. The National Rural Health Association reports that 18% of the United States of America rural populations (vs. 12% urban) are comprised of adults aged 65 and older.^9^ The most recent United States of America (USA) census shows that more than 50% of older adults in some states live in rural areas.^10^ Reasons for rising populations of older adults in rural areas in the United States of America may include attraction to retirees and movement of younger populations to urban areas for education, work, and social life.^11^ Similar movement to and from rural areas have been observed in Europe,^12^ and the World Bank and World Urbanization Prospects report rising trends of older adults in rural areas worldwide.^13^, ^14^ Further, older adults in rural areas experience higher rates of poverty and chronic illness coupled with poorer health behaviors, when compared with urban areas.^9^, ^15^

Developing an age‐friendly system requires local level engagement and support to assess needs and prioritize interventions that promote older adults' health and ability to age in place.^16^ Persons living in rural communities experience different challenges than those living in metropolitan communities regarding access to affordable housing, transportation, healthcare, and community services.^17^ Taken together, this suggests that a careful examination of rural age‐friendly systems is needed. Therefore, the purpose of this project is to conduct an international scoping review of the empiric literature on the rural age‐friendly ecosystem. This scoping review seeks to (1) map the state of the science of age‐friendly systems in rural areas regarding structural characteristics, processes for delivering age‐friendly practices, and outcomes of age‐friendly systems, (2) analyze strengths, weakness, opportunities, and threats of age‐friendly system implementation, and (3) make person, practice, and policy‐level recommendations to support active aging and development of age‐friendly communities.

## 2 METHODS

In this scoping review, we mapped the empiric literature on the rural age‐friendly ecosystem. We selected a scoping review over other types of reviews because it best aligns with the objective of summarizing the quantity and characteristics of the literature by design and other key features.^18^ We first developed a protocol for the review, which included a working objective, methods, and proposed timeline and tasks. The team included several experts in conducting reviews and used a consensus model in creating the protocol.

### 2.1 Identifying the research question

In keeping with Arksey and O′Malley methodology^19^ we began with a broad, generalist research question: “What empiric evidence is available for age‐friendly ecosystems in rural areas?” Through multiple meetings with the research team, the scope of inquiry was clarified as suggested by Levac et al.^20^ by defining the concept of age‐friendly, providing rationale for a focus on rural populations exclusively, and the outcomes of interest—studies which reported empiric data. This resulted in a refined question of “What empiric evidence exists which supports age‐friendly ecosystems in rural areas?”

### 2.2 Identifying relevant studies

In this second stage, the focus was on developing a decision plan for our literature search with an emphasis on being as comprehensive as possible. This began with a series of meetings between the team leader (H. B.) and a health sciences librarian with expertise in systematic searches to discuss the scope of the project and strategies available to answer our research question.

#### 2.2.1 Inclusion/exclusion criteria

Comprehensive inclusion criteria were agreed upon and required that any accepted papers must use an “age‐friendly” framing or model. Progenitor terms such as “elder friendly” or associated terms such as “patient priorities care” were accepted. Additional criteria included human studies, sample age of 45 years of age or older (to assure only adult studies), papers self‐identified as rural and reported empiric data (quantitative or qualitative). Studies were excluded if not using age‐friendly framing; meta‐analyses and systematic reviews were also excluded.

#### 2.2.2 Data sources and search strategy

**Figure 1:**
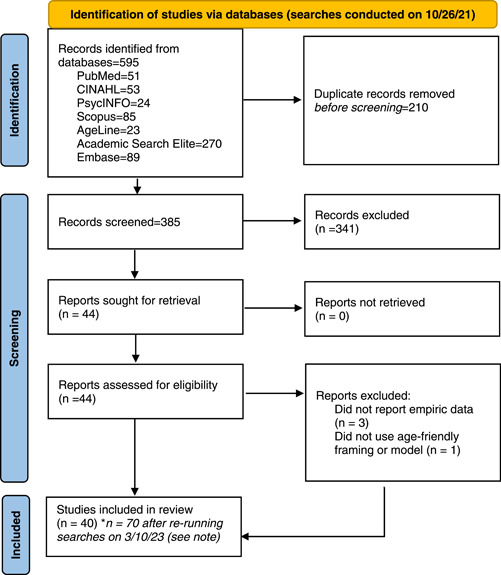
PRISMA 2020 flow diagram for identification and screening of studies. *Searches were rerun on March 10, 2023, and resulted in an additional 131 non‐duplicate articles. Of these, 30 articles were deemed eligible for inclusion. Therefore, the final number of studies included in this review were 70. Page MJ, McKenzie JE, Bossuyt PM, Boutron I, Hoffmann TC, Mulrow CD, et al. The PRISMA 2020 statement: an updated guideline for reporting systematic reviews. BMJ 2021;372:n71. doi: 10.1136/bmj.n71. For more information, visit: http://www.prisma-statement.org/.

The medical librarian conducted a series of test searches, which the team leader (H. B.) reviewed to determine the specific terms and databases to query. After this preparatory phase, searches were conducted in PubMed, CINAHL, AgeLine, PsychINFO, EMBASE, Scopus, and Academic Search Elite on October 26, 2021. The same searches were rerun on March 10, 2023 in the same databases to update the search. See Supporting Information: Table S1 for full list of terms used by database for the search. This initial search resulted in identifying 385 papers, and the second search yielded an additional 131 non‐duplicate articles. See Figure 1 for PRISMA flow diagram for disposition of the original papers.

### 2.3 Study selection

#### 2.3.1 Title and abstract

The title/abstract screening used a hybrid of human‐ and technology‐screening processes. After the team was trained on the inclusion and exclusion criteria and use of the spreadsheet for the title/abstract screen, four team members (D. L., S. T., S. M., E. G.) screened 25% of the studies per member for exclusion criteria in the human screen. One team member (L. N.) then conducted a random 20% reliability check, which resulted in 93.5% (72/77) agreement. In the technology round, term identification (e.g., methods, sample, or data) in the spreadsheet was used to identify studies meeting the inclusion criteria of empiric studies. When in doubt, a paper was retained for the next level of screening. For example, 25 papers that did not include an abstract were automatically advanced to full text screening if inclusion/exclusion criteria were not clear from the title. Inclusion criteria of age and rural were confirmed during the full text screening. A 100% reliability check was conducted on this set of papers and any discrepancies were justified during team meetings.

#### 2.3.2 Full‐text screening

Ten team members (D. L., A. K., J. B., S. T., S. M., E. G., N. C., S. J., L. N., H. B.) conducted a full text screening and data extraction (7−11 articles per team member). Once again, a 100% reliability check was conducted resulting in each paper being examined by at least two people. Effort was made to give each screener a new set of papers from their previous screen. The inclusion/exclusion criteria were applied again before any extraction took place. Forty papers met the protocol criteria and advanced to data extraction.

### 2.4 Charting the data

#### 2.4.1 Data abstraction and management

Using the study protocol, a second spreadsheet was developed which included the data elements necessary to answer our research question. The full team went through a hands‐on training session covering definitions for each data element, reviewing an example paper which had undergone full extraction, and then a practice group extraction. Five study domains (publication, study design, analysis elements, intervention elements, and study outcomes) were assessed. Subsequent, one‐on‐one training with the team leader (H. B.) was held as needed. A final abstraction quality check was conducted by the team leader (H. B.) after all data forms were submitted.

### 2.5 Collating, summarizing, and mapping the results

Following Arksey and O′Malley's methodology,^19^ we developed a meta synthesis codebook, since descriptive‐analytic techniques are used for this narrative review type of analysis.^21^ The codebook (Supporting Information: Text S3) included the working objective for the paper, and provided code names, definitions, and indicators for each code across three analytic layers. A subset of the team with qualitative experience (D. L., A. K., J. B., H. B.) met in a series of analytic sessions to identify and categorize data elements according to our analytic framework. The team plotted studies using the 4‐level socio‐ecological model (individual, interpersonal, organizational, and environmental levels).^22^ The socio‐ecological model was chosen because all the papers in the data set reported information across multiple levels. Donabedian's three component model for evaluating quality of care was used to examine characteristics of structure, process, and outcomes, given that most of the papers involved quality improvement projects.^23^ Each data element was adjudicated using Donabedian's model to identify whether the data element addressed structural characteristics, processes for delivering age‐friendly practices, or outcomes of age‐friendly systems by socio‐ecological level to identify specific gaps in evidence. Finally, a SWOT analysis allowed us to compare internal (strengths, weaknesses) and external (opportunities, threats) factors identified by articles included in this synthesis that impact implementation and sustainability of age‐friendly systems in rural areas.^24^ As a final step, the socio‐ecological model was collapsed into two categories (person vs. system‐level) to develop a clear map of the results and examine strengths, weaknesses, opportunities, and threats across these two categories.

## 3 RESULTS

### 3.1 Study characteristics

Most of the included studies (*N* = 70) were conducted in five countries: the United States of America (*n* = 23), Canada (*n* = 13), China (*n* = 7), the Netherlands (*n* = 7), and Australia (*n* = 5) (Table 1). The most common study designs were quantitative (*n* = 28), qualitative (*n* = 25), mixed methods (*n* = 8), and implementation (*n* = 8) studies. Units of analysis included older adults/adults (*n* = 41), clinicians/healthcare providers (*n* = 12), service (e.g., community) providers (*n* = 7), caregivers/family (*n* = 3), government agencies/employees (*n* = 3), age‐friendly leaders or committee members (*n* = 3), communities (*n* = 3), students (*n* = 1), and health systems (*n* = 3). Forty‐eight studies used the World Health Organization age‐friendly cities framework, 10 studies used the 4Ms framework for age‐friendly care, and 10 studies did not report a guiding framework. Specific information about study samples and purposes can be found in Table 1.

**Table 1:** Study characteristics.

Authors	Country	Study design	Sample	Study purpose	Age‐friendly framework
Ahmadi et al.^25^	Iran	Quantitative	26 hospitals	Examine age‐friendliness of public hospitals and provide background to policymakers	WHO
Arain et al.^26^	Canada	Implementation pilot study	88 clinicians	Assess usefulness of elder‐friendly care (EFC) materials and resources, staff practice changes and perceptions, and readiness for scale and spread	4Ms
Aung et al.^27^	Japan	Mixed methods	243 older adults	An age‐friendly environment in Japan was assessed through the perceptions of community residents and their interaction with the environment	WHO
Bendien et al.^28^	Netherlands	Qualitative	74 older adults	Investigate how older people experienced COVID‐19 mitigation measures and whether these measures endorse and promote the idea of an age‐friendly world	WHO
Berish et al.^29^	USA	Implementation	3 Primary care sites	To collaboratively implement the age‐friendly health systems framework, at the primary health network (PHN)	4Ms
Black and Oh^30^	USA	Qualitative	30 communities	Assess the progress reported by American age‐friendly communities	WHO
Blaum et al.^31^	USA	Implementation Pilot study	14 clinicians 119 older adults	Describe the implementation processes and feasibility of patient priorities care	PPC
Breda et al.^32^	USA	Implementation	1598 older adults	Evaluate outcomes associated with an integrated inpatient and outpatient program aimed at optimizing the care of geriatric fracture patients	4Ms
Brossoie et al.^33^	South Korea	Quantitative	1836 adults	The transferability of a US‐developed age‐friendly community resident survey, based on the WHO framework, was tested in two South Korean cities	WHO
Choi^34^	USA	Quantitative	5999 adults	Examine the interrelationship between the availability of age‐friendly features, perceived age‐friendliness of community, and intention toward AIP	WHO
Chu^35^	China	Quantitative	8075 older adults	Examine whether local governments' policy efforts on age‐friendly communities promote older adults' social participation	NR
Chui et al.^36^	China	Qualitative	65 older adults	Examine what older adults' perceptions of community shortcomings in meeting psychosocial and physical needs as they age	WHO
Chui et al.^37^	China	Mixed methods	5272	Evaluate changes in perceived age‐friendliness with community‐dwelling older adults	WHO
Chung and Kim^38^	South Korea	Quantitative	590 adults	Explore the relationship between age‐friendly environment, social support, sense of community, and loneliness	WHO
Colibaba et al.^39^	Canada	Qualitative	10 older adults	Explore older adults' perspectives on rural age‐friendly programming in a community that implemented age‐friendly community	WHO
Cramm et al.^40^	Netherlands	Mixed methods	558 older adults (quantitative), 32 older adults (qualitative)	Characterize the relationship between frailty and ageing in place; identify differences in neighborhood characteristics supporting ageing in place missed by frail and non‐frail older people	WHO
Doolan‐Noble et al.^41^	New Zealand	Workshop	133 attendees (older adults, caregivers, healthcare providers, academics, researchers, governmental, and nongovernmental)	Describe a workshop process conducted to guide funding priorities for the ageing well national science challenge in New Zealand	WHO
Emlet and Moceri^42^	USA	Qualitative	23 older adults	Explore importance of social relationships and connectedness with aging in place and in developing elder‐friendly communities	WHO
Everingham et al.^43^	Australia	Qualitative	37 older adults, service groups, providers, and governmental	Understand issues impacting on older adults' capacity to access relevant information	NR
Fang et al.^44^	Multiple	Mixed methods	192 adults	Initiate the conceptualization of an intergenerational, age‐friendly living ecosystem to enhance public health planning	WHO
Garner and Holland^45^	UK	Quantitative	132 older adults	Describe development and validation of the age‐friendly environment assessment tool and assess whether individual function and frailty impact perceptions of environmental age‐friendliness	WHO
Greenfield and Reyes^46^	USA	Qualitative	8 service providers	Derive a typology of older adults' engagement based on core group members' descriptions of how older adults are involved in their communities' initiatives	WHO
Hancock et al.^47^	Australia	Qualitative	262 older adults and family members	Explore older adults' views on what is important in maintaining health and well‐being align with the eight WHO age‐friendly domains, and which are most salient	WHO
Hanson et al.^48^	Canada	Mixed methods	30 clinicians	Inform the design and implementation of a larger initiative on elder‐friendly interventions to improve postoperative healthcare practices and health outcomes	NR
Harrison et al.^49^	UK	Qualitative	13 older adults	Adapt photo‐elicitation to explore the age‐friendliness of a rural area	WHO
Hawley et al.^50^	USA	Implementation pilot study	180 clinicians	Provide a step‐by‐step guide to workshop implementation, including all necessary materials, and results from virtual evaluation	4Ms
Hewson et al.^51^	Canada	Mixed methods	32 organization workforces	Examine service providers' readiness to address social participation expectations of aging baby boomers using an age‐friendly cities framework in a mid‐sized city	WHO
Hunter et al.^52^	Canada	Qualitative	12 clinicians	Understand safety and harm in ED for older adults with dementia from the perspective of healthcare professionals	NR
Jagroep et al.^53^	Netherlands	Quantitative	697 older adults	Examined relationships of neighborhood characteristics with physical activity	WHO
John and Gunter^54^	USA	Mixed methods	387 older adults	Gain better understanding of urban and rural contexts for place‐based aging to inform programs and policy	WHO
Kim et al.^55^	USA	Quantitative	3652 older adults	Assess the factor structure of age‐friendly indicators in the AARP AFC Surveys and evaluate the effects of these constructs on self‐rated health	WHO
Khoddam et al.^56^	Iran	Quantitative	160 older adults	Determine compatibility of city characteristics with indicators of the age‑friendly city based on WHO criteria	NR
Korte et al.^57^	USA	Qualitative	13 service providers	Assessed service providers' knowledge of older adults' medical issues and health education needs	4Ms
Lauckner and Stadnyk^58^	Canada	Qualitative	35 older adults	Examine how age friendly community consultations provide strategies for occupational therapists	WHO
Lei and Feng^59^	China	Quantitative	5641 older adults	Examine association between neighborhood environment characteristics within age‐friendly communities and depressive symptoms among older adults	WHO
Lesser et al.^60^	USA	Quantitative	1684 clinicians	Examine clinicians' attitudes, knowledge, and practices concerning the 4Ms	4Ms
Loukaitou‐Sideris et al.^61^	USA	Mixed methods	8219 households	Analyze a statewide data set and to understand travel behavior of older adults versus non‐older adults	NR
Lynch et al.^62^	USA	Quantitative	61 clinicians	Assess readiness of cancer programs to provide age friendly cancer care	WHO and 4Ms
Ma et al.^85^	Australia	Qualitative	20 service providers	Assess potential outcomes of actions to support older people's mobility within an age‐friendly city	WHO
Matei et al.^47^	Romania	Mixed methods	100 older adults, 100 carers	Determine if characteristics and services of residential care centers for elders were appropriate for older adults considering WHO guidelines	WHO
McCrillis et al.^63^	Canada	Qualitative	46 age‐friendly leaders	Address influence of unique, rural community contexts that differentially impact age‐friendly initiatives' longer‐term sustainability	NR
Menec and Nowicki^64^	Canada	Quantitative	646 younger and older adults	Examine communities' age‐friendliness and relationship to health‐related outcomes	WHO
Menec et al.^65^	Canada	Quantitative	990 residents from 39 rural communities	Examine the congruence between two types of age‐friendly surveys: subjective assessments by community residents versus objective assessments by municipal officials	WHO
Morgan et al.^66^	USA	Quantitative	3370 older adults	Analyze 5 health equity factors and receipt of age‐friendly care	4Ms
Mudge et al.^67^	Australia	Implementation	539 older adults	Implement and evaluate a ward‐based improvement program (“Eat Walk Engage”) to more consistently deliver age‐friendly principles of care	NR
Neville et al.^68^	New Zealand	Qualitative	22 stakeholders (government, older adults, and community)	Explore the barriers to communities developing age‐friendly initiatives	WHO
Nieboer and Cramm^69^	Netherlands	Quantitative	945 older adults	Identify relationships between age‐friendly environments and older adults' overall well‐being	WHO
Novek and Menec^70^	Canada	Qualitative	30 older adults	Use a participatory methodology to explore older adults' perceptions of age‐friendliness	WHO
Orpana et al.^56^	Canada	An evidence‐based, iterative consultation approach	Not reported	Develop indicators to support the evaluation of age friendly communities relevant to a wide range of Canadian communities	WHO
Özer et al.^71^	Turkey	Quantitative	306 older adults	Test the Turkish validity and reliability of the age‐friendly cities and communities questionnaire	WHO
Park and Lee^72^	South Korea	Quantitative	1657 older adults	Examine the role of environment on the well‐being of vulnerable older adults in a non‐western context using the WHO's framework for age friendly cities and examine older adults' life satisfaction	WHO
Pestine‐Stevens and Greenfield^73^	USA	Qualitative	8 service providers	Describe ways in which age‐friendly community initiatives core teams described working with other organizational entities	WHO
Plasencia^74^	USA	Qualitative	72 older adults	Examines how older Latinxs characterize age‐friendly communities	NR
Pohnert et al.^75^	USA	Implementation	1100 clinics	Describe the implementation of 4Ms framework	4Ms
Pope and Greenfield^76^	USA	Qualitative	8 age‐friendly team leads	Exploring community events as a mechanism through which practitioners work toward age‐friendly city goals	WHO
Russell et al.^77^	Canada	Qualitative	35 age‐friendly committee members	Examine the challenges and opportunities to sustaining age‐friendly programs in the Canadian age‐friendly funding program	WHO
Russell et al.^78^	Canada	Qualitative	11 age‐friendly leaders	Examine the connection between rural age‐friendly longevity and its ability to facilitate supportive change for older adults in rural communities	WHO
Shi et al.^79^	China	Quantitative	5629 older adults	Explored the effects of the age‐friendly city and community on older adults' cognitive health	WHO
Shih et al.^80^	Taiwan	Qualitative	46 clinicians and staff	Explore the difficulties faced by primary health center staff in the implementation of age‑friendly policies	Age‐friendly certification framework
Southerland et al.^81^	USA	Quantitative	66 healthcare providers	Understand how healthcare providers perceive their EHRs and to identify any current best practices and ideas for improvement regarding integration of the 4Ms	4Ms
Teixeira‐Poit^82^	USA	Qualitative	48 service providers	Understand how organizations collaborate in age‐friendly communities to provide services to older adults	WHO
Tewary et al.^83^	USA	Implementation	16 healthcare providers	Evaluate progress through six e‐clinical measures that collectively provide indicators of the 4Ms framework	4Ms
Thissen and Droogleever Fortuijn^84^	Netherlands	Quantitative	978 older adults	Investigate changes in person‐environment fit for older people in a rural area	WHO
van den Berg et al.^85^	Netherlands	Qualitative	5 older adults, 7 students	Gain insight into the experiences of older adults and undergraduate students in cocreating age‐friendly services	NR
Van Hoof et al.^86^	Netherlands	Quantitative	393 older adults	Investigate how older citizens view the age‐friendliness of their city	WHO
Walsh et al.^87^	Ireland	Qualitative	62 community stakeholders	Explore the role of informal practices, across the state, private, voluntary, and family and friends' systems, in addressing age‐friendly concept in rural communities	WHO
Wang et al.^88^	China	Quantitative	301 rural villages, 152 urban communities	Analyze community‐level measures in the China Health and Retirement Longitudinal Study within the framework of WHO's age‐friendly cities and compare the age‐friendliness between rural and urban settings	WHO
Winterton^89^	Australia	Qualitative	26 community service stakeholders	Explore barriers experienced by diverse rural community stakeholders in facilitating environments that enable age‐friendly social participation	WHO
Yu et al.^90^	China	Quantitative	863 older adults	Investigate age‐friendly rural communities' impact on the quality of life of older adults	WHO
Zhang et al.^91^	USA	Mixed methods	Not reported	Examine if the physical, built, and social environments differentiate communities with better community health across the rural–urban divide	WHO

Abbreviations: NR, none reported; PPC, patient priorities care; WHO, World Health Organization age‐friendly cities framework; 4Ms, 4Ms framework for age‐friendly care.

### 3.2 Structural factors, processes, and outcomes of age‐friendly systems in rural ecosystems

**Figure 2:**
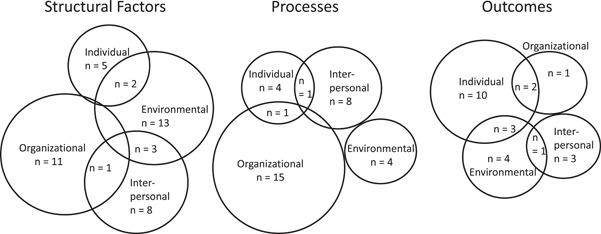
Structural factors, processes, and outcomes of age‐friendly systems.

Figure 2 maps existing evidence related to the structural factors, processes, and outcomes of age‐friendly systems in rural empiric literature at the *individual, interpersonal, organizational*, and *environmental* levels. Supporting Information: Table S2 contains further details regarding study data.

#### 3.2.1 Structural factors

Evidence related to the structural factors of age‐friendly systems included a focus on: (1) *individual* attitudes^54^, ^86^, ^92^ and barriers^40^, ^43^ for age‐friendly systems; (2) *interpersonal* connectedness,^42^ community,^63^, ^70^ and participation^52^, ^89^ in age‐friendly systems; (3) *organizational* integration and planning,^39^, ^42^, ^48^, ^51^, ^91^, ^93^ characteristics,^47^, ^48^ collaboration,^82^ funding^41^, ^77^, ^78^; and (4) *environmental* fit,^36^, ^40^, ^70^ transportation and access,^61^, ^85^, ^87^, ^89^, ^92^ jurisdiction,^63^ and built environment.^34^, ^37^, ^44^, ^56^, ^89^

#### 3.2.2 Processes

Evidence related to the process of delivering age‐friendly practices included a focus on: (1) *individual* roles^48^, ^87^ and identification of older adult priorities^31^, ^49^; (2) *interpersonal* communication, collaboration, and partnership^26^, ^30^, ^48^, ^77^, ^94^; (3) *organizational* workflow,^29^, ^31^, ^80^ electronic health records,^81^ congruence/readiness,^58^, ^62^, ^88^ staff training, preparedness,^25^, ^50^, ^80^ and engagement,^73^, ^76^ and evaluation^65^, ^66^; and (4) *environmental* evaluation^45^, ^55^ and barriers or challenges.^68^, ^84^

#### 3.2.3 Outcomes

Evidence related to outcomes of age‐friendly systems largely focused on improving individual and community health and related outcomes, including persons' quality of life,^45^, ^90^ life satisfaction,^64^, ^72^ perceived health,^64^ well‐being,^69^, ^92^ community health,^91^, ^92^ physical activity,^53^, ^85^ cognitive health,^79^ civic/social participation, engagement, and support,^27^, ^35^, ^38^ and levels of depression^59^ and loneliness,^45^ while the mechanism of improving those outcomes exist on multiple levels (individual, interpersonal, organizational, environmental) (Figure 2).

### 3.3 SWOT analysis of age‐friendly system implementation and sustainability in rural areas

Strengths, weaknesses, opportunities, and threats of age‐friendly system implementation and sustainability in rural areas are summarized at the *person* and *system* level (Table 2).

**Table 2:** SWOT analysis of age‐friendly system implementation and sustainability in rural areas.

Domains	Strengths	Weaknesses	Opportunities	Threats
Person	Positive impact on quality of life,^73^, ^90^ life satisfaction,^64^, ^72^ cognitive health,^79^ physical activity,^53^ civic/social participation and engagement,^27^, ^35^ perceived health,^64^ and lower depression^59^ and loneliness^73^ Facilitates clinician identification of patient priorities,^55^ family care planning,^60^ and family contributions to dementia care^80^ Individual well‐being^53^ and social support^38^ positively impacted by AFS neighborhood characteristics AFS resulted in reduced length of stay, direct costs,^32^ and delirium incidence^67^	Higher levels of community support associated with lower life satisfaction among poor older adults living alone^72^ Volunteer burnout^71^ Social connectedness,^86^ communication, teamwork, leadership^35^, ^71^ important	Important for older persons with frailty^79^ Informal practices such as collective interdependencies and roles strengthen capacity to enhance age‐friendliness^57^ Sense of community is a sustainability facilitator^81^ Consider cocreation with older adults^46^, ^85^ Photo‐elicitation as opportunity to promote AFS development^57^ Clinicians acknowledge benefits of providing care within AFS^60^ Cross‐cultural considerations in AFS^74^ and age‐friendly surveys^33^, ^71^	Perceptions of age‐friendliness^27^, ^86^ and individual acceptance of new roles and duties^35^ vary Barriers to older adults accessing information^67^ Community champions and partnerships important for sustainability^71^ Limited funding to support social participation^33^ Service providers need education to support older adults^57^
System	Community health,^68^ sense of community,^38^ and well‐being positively impacted by neighborhood characteristics, such as transportation and access to outdoor/indoor spaces^84^ Built environment (housing, transportation) impact quality of life,^90^ life satisfaction, and self‐perceived health^64^ Exercise and recreational facilities associated with lower depression^59^ Validated tool for AFS environment assessment^55^, ^73^ and community level indicators for AFS develped^53^ AFS resulted in reduced length of stay and direct costs^32^ Age‐friendly features associated with perceived age‐friendliness of community^46^, ^57^ AFS result in improved medication management, advanced care planning, and fall risk assessment^83^	Dependence on volunteers^32^, ^71^ and staff training^81^ Issues of scope, reach, and sustainability^79^ Common AFS survey overestimated communities age‐friendliness^33^ Community characteristics did not impact life satisfaction and self‐perceived health^64^ Rural/urban divide in environmental impact^68^	Integrate AFS into existing structures^86^ and funding priorities^67^ Congruence between existing practices and AFS^86^ Increase clinician preparedness for AFS with workshop^83^ Accessibility and informal practices underpin community responses to supporting older persons^57^ Community history and identity impact AFS^83^ Walkability and transportation available in communities^35^ Incorporation of 4Ms into the annual wellness visit^67^ Implementation strategies for AFS^75^, ^80^ Community events to boost older adult engagement in AFS^86^ Organizations can work together in AFS^73^	Education, environment, staffing, policies, and other research projects as factors influencing organizational readiness for change^5^ Community planning critical^68^ Community barriers: getting started, minimal diversity,^68^ financial constraints,^32^, ^71^ collaboration between organizations,^27^ Jurisdictional fragmentation^81^ Readiness of service providers to meet the emerging social participation needs of older persons^46^ Health system barriers: readiness of healthcare system,^79^ electronic health record,^81^ workflow challenges: limited time, poor communication with specialists, reimbursement challenges^7^ and health equity issues^71^ Challenges in implementing AFS in poorer communities^84^ and WHO organizational domains do not apply well in developing areas^80^ Environment needs to adapt to older adults^46^: pedestrian crossings, special queues,^53^ buildings and transportation^33^, ^85^ AFS should include sensory, physical, and sociocultural factors^53^ COVID‐19 presented new barriers to AFS^28^

Abbreviations: AFS, age‐friendly system; WHO, World Health Organization.

#### 3.3.1 Strengths

Main strengths of age‐friendly systems identified at the *person* level were the impact on individual outcomes^27^, ^32^, ^35^, ^38^, ^45^, ^53^, ^59^, ^64^, ^67^, ^69^, ^79^, ^90^ and incorporating patient priorities^31^ and family in care.^26^, ^52^ Main strengths of age‐friendly systems identified at the *system* level was the impact of built environment (including transportation and recreational facilities) and organizational age‐friendly processes on persons' outcomes.^59^, ^64^, ^83^, ^90^, ^91^, ^92^

#### 3.3.2 Weaknesses

Main weaknesses of age‐friendly systems identified at the *person* level were issues with volunteer burnout^77^ and importance of social connectedness,^42^ communication, teamwork, and leadership.^30^, ^48^ Main weaknesses of age‐friendly systems identified at the *system* level were dependence on volunteers^77^, ^78^ and staff training,^25^ rural/urban divide in environmental impact,^91^ and issues of scope, reach, and sustainability.^39^

#### 3.3.3 Opportunities

Main opportunities for age‐friendly systems identified at the *person* level were existing sense of community,^63^ cross‐cultural considerations,^33^, ^71^, ^74^ fit with existing clinician goals or practices,^60^, ^87^ and cocreation with older adults.^46^, ^49^, ^94^ Main opportunities for age‐friendly systems identified at the *system* level were integration of age‐friendly systems into existing structure and practices,^29^, ^41^, ^42^, ^58^, ^75^ existing community features,^61^, ^70^, ^87^ and workshop/community engagement opportunities.^50^, ^73^, ^76^

#### 3.3.4 Threats

Main threats to age‐friendly systems identified at the *person* level were differences in individual perceptions and acceptance of roles and importance^48^, ^54^, ^86^ of champions (e.g., advocates) and partnerships^77^ and need to educate service providers to support older adults.^57^ Main threats to age‐friendly systems identified at the *system* level were readiness for change,^48^, ^51^ adapting to needs of older adults,^36^, ^56^, ^85^, ^89^ community^44^, ^63^, ^68^, ^77^, ^78^, ^82^ and health system^31^, ^62^, ^66^, ^81^ barriers, challenges in poor/developing areas,^84^, ^88^ and additional COVID‐19 related barriers.^28^

### 3.4 Summary

Three key takeaways became clear from this qualitative synthesis. First, age‐friendly systems are heterogeneous, involving acute care to community settings and multiple frameworks. However, there are unifying elements, such as a focus on encouraging active aging and a few prominent models: the World Health Organization age‐friendly cities framework and the 4Ms framework for age‐friendly care. Articles in this review focused on age‐friendliness of a variety of systems in rural areas, including hospitals, hospital units, cities, communities, community service agencies, and residential care centers. While articles included a variety of stakeholders, such as service (e.g., community) providers, caregivers/family, government agencies/employees, age‐friendly leaders or committee members, communities, students, and hospitals, the majority focused on older adults and clinicians/healthcare providers. The structure, process, outcomes map revealed that most evidence to date has focused on structural factors of age‐friendly health systems, followed by processes for delivering age‐friendly practices. Least common was data on outcomes of age‐friendly systems in the rural ecosystem, especially at the interpersonal and organizational level. While the SWOT analysis revealed many strengths, including its impact on persons' outcomes,^27^, ^32^, ^45^, ^53^, ^59^, ^64^, ^67^, ^69^, ^79^, ^90^ and opportunities for age‐friendly systems in rural areas, it also revealed several weaknesses, threats, and gaps.

## 4 DISCUSSION

The purpose of this review was to map the state of the science on rural age‐friendly systems and make person, practice, and policy‐level recommendations to support active aging and development of age‐friendly communities. Overall, much work remains to be done in this important area. The unifying element in this scoping review was the focus on the rural age‐friendly ecosystem. Further, it was discovered that while many terms and places were identified in this review, age‐friendliness was framed in similar ways. The most common framework for age‐friendliness in articles in this review was the World Health Organization age‐friendly cities framework, which has 8 domains: community and healthcare, transportation, housing, social participation, outdoor spaces and buildings, respect and social inclusion, civic participation and employment, communication, and information.^95^ Several articles alternatively used the 4Ms framework for age‐friendly care, which focuses on four evidence‐based elements of high quality care: what matters, mobility, mentation, and medication.^96^, ^97^, ^98^ It appears appropriate that articles focused on age‐friendly communities or cities would fit more closely with the World Health Organization age‐friendly cities framework and those focused on healthcare systems would fit the 4Ms framework for age‐friendly care.

### 4.1 Critical gaps in the evidence

This review reveals some limitations of existing empiric literature on the rural age‐friendly ecosystem. While articles included a variety of stakeholders, the significant majority focused on older adults and clinicians/healthcare providers. This focus fits the purpose of improving care for older persons and clinicians/healthcare providers being positioned to impact this movement. However, we found limited data on caregiver and family involvement in age‐friendly systems and its impact on their outcomes/experience. This gap is critical as supporting family caregivers is a key aspect of age‐friendly acute and community care.^99^, ^100^ Further, our structure, process, outcome map revealed limited outcomes relevant to organizations, such as return on investment or healthcare utilization. This gap may impede advocating for implementation and sustainability of age‐friendly systems in an organization. Last, publications from only five countries were identified in this review, which could reflect limitations of the literature searches conducted for this review or of the overall evidence base.

### 4.2 System level weaknesses and threats

Because most of the articles in this review involved quality improvement projects, we completed a SWOT analysis of age‐friendly system implementation and sustainability in rural areas. While the SWOT analysis revealed many strengths, it also revealed several weaknesses, threats, and gaps. Age‐friendly systems have weaknesses due to reliance on specially‐trained volunteers^77^, ^78^ and staff,^25^ communication, and teamwork.^30^, ^48^ Therefore, if a health system or community is lacking special age‐friendly training or strong existing communication or teamwork, implementation of age‐friendly practices may be unsuccessful. Many system‐level threats to age‐friendly system implementation and sustainability were identified, including community^44^, ^63^, ^68^, ^77^, ^78^, ^82^ and health system^31^, ^62^, ^66^, ^81^ barriers, additional COVID‐19 related barriers,^28^ and challenges in low resource areas.^84^, ^88^ Only a few studies in this review focused on age‐friendly systems in low resource areas, and they identified specific challenges.

### 4.3 Recommendations

#### 4.3.1 Person

Development of age‐friendly systems and the associated evidence base present an opportunity to align healthcare and communities with needs and priorities of the older adult population in rural ecosystems. Evidence suggests that age‐friendly systems have a positive impact on patient and community outcomes, including quality of life,^45^, ^90^ life satisfaction,^64^, ^72^ perceived health,^64^ community health and well‐being,^91^ physical activity,^53^ and lower depression^59^ and loneliness.^45^ Therefore, we recommend working towards implementation of age‐friendly systems in rural areas. Important foci of future work should include addressing gaps in evidence, including involvement of family caregivers in age‐friendly systems and impact of age‐friendly systems on organizational outcomes, such as return on investment and healthcare utilization.

#### 4.3.2 Practice

Challenges for implementation and sustainability of age‐friendly systems in practice include often limited funding and differences in impact observed in urban versus rural areas.^91^ To address funding limitations, one article in this review describes opportunities for setting funding priorities around age‐friendly systems.^41^ As the current state of the science is largely limited to documentation of these weaknesses and threats for age‐friendly systems, future work should focus on opportunities to address or avoid such challenges, such as cost considerations, promoting readiness in organizations, preparing the workforce, considering paid staff versus volunteers, and adapting to needs and what matters to the older person and family caregivers. For example, it is important to examine cost of implementing and sustaining age‐friendly systems against potential return on investment or cost savings at an organizational or public health level.

Implementation of age‐friendly systems is an important public health issue impacting the health and well‐being of our older adult populations.^101^ There were approximately 54 million adults age 65 and older in the United States as of 2021, and the number is expected to reach over 80 million by 2050.^102^, ^103^ Adding to this concern is actual and projected increase in the old age dependency ratio worldwide, which indicates increasing proportion of individuals age 65 per individuals who are in the working age population (e.g., 15−64).^104^ These trends, combined with higher disease burden^105^ and functional decline^106^ in older adults, contributes to concern about infrastructure and resources available to meet the needs of our aging populations. Viewing these challenges through a public health lens presents an opportunity for connecting and coordinating sectors and professions that provide services and infrastructure to promote healthy aging, increase access, and identify gaps.^101^ To increase support for age‐friendly systems, this review reveals the need for more data and information on implementing age‐friendly systems across institutions and communities in the rural ecosystem and the impact of various types of age‐friendly systems at a person and system level.

#### 4.3.3 Policy

Policy recommendations of this work begin with the need to promote opportunities to leverage improvements in age‐friendliness at a community level. Age‐friendly systems are commonly established and maintained at the community level, so that interventions fit with local needs. One challenge with both increasing older adults' access to existing community services and increasing collaboration or shared services between rural communities is transportation and availability of transportation services.^107^ Policy focused on increasing age‐friendly transportation infrastructure is needed to improve community connections among rural older adults. In addition, improvements in rural technology infrastructure can assist connecting older adults to existing community and healthcare services. This review identified specific challenges in low resource areas. Therefore, future research should consider cost/funding, existing infrastructure, and specific potential benefits of age‐friendly systems in low resource areas.

### 4.4 Limitations

Our literature search was limited to articles published in English only. The decision to preclude a “gray” literature or a comprehensive bibliography review was made given the involvement of an expert health sciences librarian, multiple test searches, use of multiple databases, and detailed inclusion/exclusion criteria. In addition, we targeted main sources and types of evidence to identify key concepts in the body of literature.^108^ Therefore, inclusion of other less rigorous sources of information were deemed more likely to yield additional early work (before publication in a peer‐reviewed journal) that would not significantly impact findings of this review. Choice of search terms and inclusion/exclusion criteria could also be considered a limitation, as some articles relevant to our purpose might have been missed. For example, publications from only five countries were identified in this review, which could reflect limitations of the literature searches conducted for this review or of the overall evidence base. However, we used a variety of progenitor terms related to “age‐friendly,” and this process was completed with a health sciences librarian with expertise in systematic searches.

## 5 CONCLUSIONS

This international scoping review maps the science on rural age‐friendly systems and makes person, practice, and policy‐level recommendations to support active aging and development of age‐friendly communities. While age‐friendly systems in this review were heterogeneous, there is an opportunity to focus on unifying elements including the World Health Organization age‐friendly cities framework or 4Ms framework for age‐friendly care. This article summarizes many potential barriers (community and health system barriers, readiness for change, adapting to needs of older adults) and facilitators (integration of age‐friendly systems into existing structure and practices) to consider when implementing and sustaining an age‐friendly system in rural areas. Despite the many potential benefits of age‐friendly systems in healthcare systems and communities, we must acknowledge limitations of the evidence base, pursue opportunities to examine organizational metrics to support implementation and sustainability of age‐friendly systems, and leverage improvements in age‐friendliness at a community level.

## AUTHOR CONTRIBUTIONS

**Daniel Liebzeit**: Conceptualization; data curation; formal analysis; investigation; methodology; visualization; writing—original draft; writing—review and editing. **Anna Krupp**: Data curation; formal analysis; investigation; visualization; writing—original draft; writing—review and editing. **Jacinda Bunch**: Data curation; formal analysis; investigation; visualization; writing—original draft; writing—review and editing. **Shalome Tonelli**: Data curation; formal analysis; investigation; visualization; writing—original draft; writing—review and editing. **Emily Griffin**: Data curation; formal analysis; investigation; visualization; writing—original draft; writing—review and editing. **Sarah McVeigh**: Data curation; formal analysis; investigation; visualization; writing—original draft; writing—review and editing. **Nai‐Ching Chi**: Data curation; formal analysis; investigation; visualization; writing—original draft; writing—review and editing. **Saida Jaboob**: Data curation; formal analysis; investigation; visualization; writing—original draft; writing—review and editing. **Lynn Nakad**: Data curation; formal analysis; investigation; visualization; writing—original draft; writing—review and editing. **Alicia I. Arbaje**: Formal analysis; investigation; writing—original draft; writing—review and editing. **Harleah Buck**: Conceptualization; data curation; formal analysis; investigation; methodology; supervision; visualization; writing—original draft; writing—review and editing.

## CONFLICT OF INTEREST STATEMENT

The authors declare no conflict of interest.

## TRANSPARENCY STATEMENT

The lead author Daniel Liebzeit affirms that this manuscript is an honest, accurate, and transparent account of the study being reported; that no important aspects of the study have been omitted; and that any discrepancies from the study as planned (and, if relevant, registered) have been explained.

## ETHICS STATEMENT

This project did not involve human participants. This research was carried out in accordance with recognized standards for scoping reviews.^18^, ^19^

## Supporting information

Supporting information.

## Data Availability

We have included relevant protocol/codebook as supplementary material for our scoping review.
