# Supplementary material for: Rural age‐friendly ecosystems for older adults: An international scoping review with recommendations to support age‐friendly communities
Source: Health Sci Rep. 2023 May 5;6(5):e1241. doi: 10.1002/hsr2.1241 (PMC10162383; doi:10.1002/hsr2.1241)
Supplement: Supplementary file 1 — Supporting information. [file HSR2-6-e1241-s001.docx]

**Supplementary Table S1**

*Search Strategy with Terms*

| **Database** | **Search Strategy** | **10/26/21 Results** |
| --- | --- | --- |
| PubMed | (patient priorities care [Title/Abstract] OR age friendly[Title/Abstract] OR age friendliness[Title/Abstract] OR elder friendly [Title/Abstract] OR AFHS [TItle/Abstract] OR 4 Ms [Title/Abstract] OR 4 M framework [Title/Abstract]) AND (rural [Title/Abstract] OR non urban [Title/Abstract] OR remote [Title] OR "Rural Population"[Mesh] OR "Rural Health Services"[Mesh] OR "Rural Health"[Mesh]) AND english[Filter] | 51 |
| CINAHL | TX ("age friendly" or "age friendliness" or "patient priorities care" OR "elder friendly" OR age N5 friendl* OR AFHS OR “4 ms” OR “4 M framework”) AND (TX rural OR TX "non urban" OR TX remote OR MH "Rural Health Centers" OR MH "Rural Population" OR MH "Rural Areas" OR MH "Rural Health Services" OR MH "Rural Health Nursing")  Filter applied: English language | 53 |
| AgeLine | TX (“age friendly” or “age friendliness” or "patient priorities care" OR "elder friendly" OR age N5 friendl* OR AFHS OR “4 ms” OR “4 M framework”) AND (TX rural OR TX "non urban" OR TX remote OR DE "Rural")  No filters applied | 22 |
| PsycINFO | TX (“age friendly” or “age friendliness” or "patient priorities care" OR "elder friendly" OR age N5 friendl* OR AFHS OR “4 ms” OR “4 M framework”) AND (TX rural OR TX "non urban" OR TX remote OR DE "Rural")  No filters applied | 24 |
| Embase | ('age friendly' OR 'age friendliness' OR 'elder friendly' OR 'patient priorities care' OR afhs OR 'age n/5 friendly' OR 'age n/5 friendliness' OR '4ms' OR '4 m framework') AND (rural OR 'non urban' OR remote OR 'rural population'/exp)  Filter applied: English language | 89 |
| Scopus | ("age friendly" OR "age friendliness" OR "elder friendly" OR "patient priorities care" OR age W/5 friendl* OR AFHS OR “4 ms” OR “4 M framework”) AND (rural OR "non urban" OR remote)  Filter applied: English language | 85 |
| Academic Search Elite | TI (“age friendly” or “age friendliness” or "patient priorities care" OR "elder friendly" OR age N5 friendl*  OR AFHS OR “4 ms” OR “4 M framework”) OR AB ( “age friendly” or “age friendliness” or "patient priorities care" OR "elder friendly" ) AND TX ( rural or "non urban" OR remote) NOT SO journal of african history  Filters applied: peer reviewed and English language | 270 |

*Note*. Search strategies were developed by the primary (DL) and mentoring authors (HB) and a health sciences librarian with expertise in systematic searching. Exact strategies comprised of both index and keyword terms appear in table below. In the interest of ensuring a comprehensive yield, filters were used sparingly without publication date restrictions (from database inception to search run date).

**Supplementary Table S2**

*Structure, Process, Outcome by Domain Map*

| Domains | Structure | Process | Outcome |
| --- | --- | --- | --- |
| Individual | -Perceptions of community availability and importance of AFS^1,66^  -Frail person-environment fit in AFS^2^  -Individual-level activities, attitudes, and capacities important in AFS^3^  -Barriers to older adults accessing information^4^ | -Individual level factors (acceptance of roles and duties) needed for AFS^5^  -Informal practices such as collective interdependencies and roles strengthen capacity to enhance age-friendliness^6^  -AFS program resulted in patient priorities identified^7^  -Photo-elicitation to promote AFS development with older adults^51^ | -Impact on quality of life^8^ and cognitive health^62^ in AFS  -Age-friendliness positively related to life satisfaction and perceived health^9^  -Individual well-being and neighborhood levels of AFS positively related^10^  -Individual perceptions of greater AFS associated with lower depression^11^  -Individual life satisfaction associated with WHO AFS domains^12^  -Age-friendliness positively related to quality of life and negatively related to loneliness^13^  -AFS impact on civic participation and engagement^41^ and physical activity^52^  -AFS resulted in reduced length of stay, direct costs,^44^ and delirium incidence^58^  -AFS result in improved medication management, advanced care planning, and fall risk assessment^65^ |
| Interpersonal | -Social connectedness is important for AFS^14^  -Sense of community as sustainability facilitator^15^  -Limited funding to support social participation for older adults^16^  -Contributions of family to AFS in dementia^17^  -AFS community impact factors: history, identity^18^  -Cross-cultural considerations in AFS^59^ and age-friendly surveys^68,69^ | -Communication, teamwork, leadership needed in AFS^5,43^  -Co-creation of AFS with older adults^19, 50^  -Utilization of AFS elements (family care planning) by acute care staff^20^  -Volunteer burnout, community champions, and partnerships important for AFS sustainability^21^  -Service providers need education to support older adults^54^ | -Neighborhood AFS characteristics positively impact community health^22^ and social participation^46^, social support, and sense of community^48^ |
| Organizational | -Education, environment, staffing, policies and other research projects as factors influencing organizational readiness for change^5^  -Integration of AFS into existing structures^14^  -Community planning critical factor to AFS^22^  -Community level indicators for AFS develped^23^  -Service providers perspectives elicited for social participation in AFS^24^  -Assess age-friendliness of characteristics and services of residential care centers^25^  -Challenges for collaboration between organizations in age-friendly community^26^  -Financial constraints and over dependence on volunteers limits AFS^21,27^  -Issues of scope, reach and sustainability of AFS^28^  -National AFS funding priorities developed^29^ | -AFS implementation identified workflow challenges^7,63^ and electronic health records barriers^64^  -Congruence between existing practices and AFS^30^  -Staff training as necessary for AFS^31,63^  -Increase clinician preparedness for AFS with workshop^32^  -Clinicians acknowledge benefits of providing care within AFS^55^  -WHO organizational domains do not apply well in developing areas^33^  -Common AFS survey overestimated communities age-friendliness^34^  -Readiness of current health care system to implement AFS^35^  -Incorporation of 4Ms into the annual wellness visit^42^  -Health equity issues in AFS^57^  -Implementation strategies for AFS^60, 63^  -Community events to boost older adult engagement in AFS^61^  -COVID-19 presented new barriers to AFS^67^  -Organizations can work together in AFS^70^ | -Community and health services as important for health and wellbeing^3^  -AFS resulted in reduced length of stay and direct costs^44^  -AFS result in improved medication management, advanced care planning, and fall risk assessment^65^ |
| Environmental | -Frailty accommodation must be part of person-environment fit in AFS^2^  -Transportation and access to outdoor/indoor spaces impact AFS^3,56^  -Accessibility and informal practices underpin community responses to supporting older persons^6^  -Jurisdictional fragmentation challenged sustainable implementation^15^  -Buildings and transportation as barriers to social participation^16^  -Community history and identity impact AFS^18^  -Transportation needs in AFS^36^  -Environment needs to adapt to older adults to be AFS^37^  -Elements in built environment- ped crossings, special queues limit AFS^38^  -Age-friendly features associated with perceived age-friendliness of community^45, 47^  -AFS should include sensory, physical, and socio-cultural factors^49^ | -Instrument validation for AFS^13, 53^  -Barriers to AFS: getting started, minimal diversity^39^  -Challenges in implementing AFS in poorer communities^40^ | -Elements of AFS built environment (housing, transportation) impact quality of life^8^  -Physical environment not community characteristics impacted life satisfaction and self-perceived health^9^  -Exercise and recreational facilities associated with lower depression^11^  -Rural/urban divide in environmental impact^22^ |

*Note*. AFS = age-friendly system; WHO = World Health Organization; ^1^John & Gunter, 2016; ^2^Cramm et al., 2018; ^3^Hancock et al., 2019; ^4^Everingham et al., 2009; ^5^Hanson et al., 2017; ^6^Walsh et al., 2014; ^7^Blaum et al., 2018; ^8^Yu et al., 2021; ^9^Menec & Nowicki, 2014; ^10^Nieboer & Cramm, 2018; ^11^Lei & Feng, 2021; ^12^Park & Lee, 2017; ^13^Garner & Holland, 2020; ^14^Emlet & Moceri, 2012; ^15^McCrillis et al., 2021; ^16^Winterton, 2016; ^17^Hunter et al., 2017; ^18^Novek & Menec, 2014; ^19^van den Berg et al., 2019; ^20^Arain et al., 2020; ^21^Russell et al., 2019; ^22^Zhang et al., 2020; ^23^Orpana et al., 2016; ^24^Hewson et al., 2018; ^25^Matei et al., 2018; ^26^Teixeira-Poit, 2020; ^27^Russell et al., 2021; ^28^Colibaba et al., 2020; ^29^Doolan-Noble et al., 2019; ^30^Lauckner & Stadnyk, 2014; ^31^Ahmadi et al., 2015; ^32^Hawley et al., 2021; ^33^Wang et al., 2017; ^34^Menec et al., 2016; ^35^Lynch et al., 2021; ^36^Loukaitou-Sideris et al., 2019; ^37^Chui et al., 2019; ^38^Khoddam et al., 2020; ^39^Neville et al., 2021; ^40^Thissen & Fortuijn, 2021^41^Aung et al., 2022; ^42^Berish et al., 2023; ^43^Black and Oh, 2022; ^44^Breda et al., 2023; ^45^Choi, 2022; ^46^Chu and Zhang, 2022; ^47^Chui et al., 2022; ^48^Chung and Kim, 2022; ^49^Fang et al., 2023; ^50^Greenfield and Reyes, 2022; ^51^Harrison et al., 2021; ^52^Jagroep et al., 2022; ^53^Kim et al., 2022; ^54^Korte et al., 2022; ^55^Lesser et al., 2022; ^56^Ma et al., 2022; ^57^Morgan et al., 2022; ^58^Mudge et al., 2022; ^59^Plasencia 2022; ^60^Pohnert et al., 2023; ^61^Pope and Greenfield 2022; ^62^Shi et al., 2023; ^63^Shih et al., 2022; ^64^Southerland et al., 2022; ^65^Tewary et al., 2022; ^66^Van Hoof et al., 2022; ^67^Bendien et al., 2023; ^68^Brossoie et al., 2022; ^69^Özer et al., 2023; ^70^Pestine-Stevens & Greenfield, 2022.

# **Supplementary Text S3**

# Codebook for

# Age-Friendly Eco-system/Patient Priorities Care Scoping Review Paper

**Working objective of this paper:** The purpose of this project is to conduct a scoping review of the international empiric literature on the rural age-friendly ecosystem. This scoping review seeks to map the state of the science, including evidence related to structure, process, and outcomes and strengths, weakness, opportunities, and threats of age-friendly systems in rural areas.

**Coding structure:**

***Analytic Layer I (numbers 1)***

Code names, definitions, and indicators are derived from the Socio-ecological model from the Centers for Disease Control and Prevention (2007) which includes individual, interpersonal, organizational, and environmental domains. This allows us to identify an accurate epistemic space to map the studies.

1. Socioecological model: these domains set up a nested yet vertical structure allowing an examination of concurrent and multi-level elements.

A. Individual level

Definition: Study findings or elements focus on individual level factors

Includes factors such as:

1) patient reported outcomes in quantitative studies

2) first person experience in qualitative studies

B. Interpersonal level

Definition: Study findings or elements focus on groups of individuals

Includes factors such as:

1) education programs

2) clubs

C. Organizational level

Definition: Study findings or elements focus on systems levels factors

Includes factors such as:

1) power structures

2) community/national levels regulations, guidelines, laws

D. Environmental level

Definition: Study findings or elements focus on tangible, large scale but non-organizational factors

Includes factors such as:

1) built environment

2) access to food, care, community

***Analytic Layer II (numbers 2)***

Since most of these papers involved quality improvement projects applying a quality care measurement analytic framework such as Donabedian’s model was deemed appropriate. Code names, definitions, and indicators are derived from Donabedian’s (2002) three component approach for evaluating quality of care which measures structures, processes, and outcomes

2. Donabedian’s Framework

A. Structure

Definitions: Study findings or elements which focus on physical and organizational characteristics of the age-friendly systems.

Includes factors such as:

1) Community features

2) Person-environment fit

B. Process

Definitions: Study findings or elements which focus on care delivered (services, treatments) by age-friendly systems. These findings or elements reflect the way the systems and processes work to deliver the desired outcome.

Includes factors such as:

1) Age-friendly systems interpersonal practices

2) Age-friendly systems roll out

C. Outcomes

Definitions: Study findings or elements which focus on effect of age-friendly systems on individual or populations

Includes factors such as:

1) Patient outcomes

2) Reported quality metrics

***Analytic Layer III (numbers 3)***

3. SWOT Analysis

A SWOT analysis was used to compare internal (strengths, weaknesses) and external (opportunities, threats) factors identified by articles included in this synthesis that impact implementation and sustainability of age-friendly systems in rural areas. Code names, definitions, and indicators are derived from Humphrey’s SWOT matrix.

A. Internal factors

1) Strengths: strong points of implementing/sustaining age-friendly systems

2) Weaknesses: weak points of implementing/ sustaining age-friendly systems

B. External factors

1) Opportunities: external to the age-friendly system, factors that represent opportunities for implementing/ sustaining age-friendly systems

2) Threats: external to the age-friendly system, factors that represent threats for implementing/ sustaining age-friendly systems
